# Psychological impact of COVID19 outbreak and coping strategies among Tunisian medical students

**DOI:** 10.1192/j.eurpsy.2022.677

**Published:** 2022-09-01

**Authors:** M. Shiri, M. Bejar, A. Arous, I. Kammoun

**Affiliations:** 1 Razi hospital, Psychiatry B, Manouba, Tunisia; 2 CHU Hedi CHaker hospital Sfax Tunisia, Department Of Psychiatry (a), Sfax, Tunisia; 3 Razi Hospital, Avicenne Psychiatry Department, Mannouba, Tunisia

**Keywords:** Anxiety and depression, medical students, covid 19, coping strategies

## Abstract

**Introduction:**

The COVID19 pandemic came with unprecedented measures that impacted every aspect of the student’s life making them vulnerable to psychological distress.

**Objectives:**

The aim of this study was to assess anxiety and depressive symptoms in relation to the coping strategies during the COVID19 pandemic among medical students.

**Methods:**

We conducted a web-based cross-sectional study among Tunisian medical students. We used an anonymous survey comprising sociodemographic characteristics, the Hospital Anxiety and Depression scale and the brief COPE.

**Results:**

A total of 216 students participated in the study; 78% were female and most respondents were enrolled in first and second year of medical studies (53%). The frequency of depressive and anxiety symptoms were respectively 28% and 40%; females experienced significantly higher depression and anxiety scores (p<10¯³ and p=0.02 respectively). Most used coping strategies were self-blame, planning, acceptance, distraction, positive reframing, active coping and religion (99%-96%). The least used coping strategies were substance use (13%) and denial (52%). We found that gender was associated with a significant difference in the coping styles where females scored higher on religious coping and denial whereas males used more humor coping. Depression and anxiety were significantly associated with denial coping. Humor, acceptance, positive reframing and active coping were significantly associated with lower depression rates. Humor coping and active coping were associated with lower anxiety rates.

**Conclusions:**

Our study shows alarming rates of depression and anxiety among Tunisian medical students during the COVID19 pandemic. A targeted intervention to promote mental health using the coping styles might be useful in this population.

**Disclosure:**

No significant relationships.

